# “Who to Tell, How and When?”: Development and Preliminary Feasibility of an Empowerment Intervention for People Living with Dementia Who are Fearful of Disclosing Their Diagnosis

**DOI:** 10.2147/CIA.S257317

**Published:** 2020-08-14

**Authors:** Jem Bhatt, Tamatha Ophelia Ruffell, Katrina Scior, Georgina Charlesworth

**Affiliations:** 1 Research Department of Clinical, Educational and Health Psychology, University College London, London, UK; 2 Research and Development, North East London Foundation Trust, London, UK

**Keywords:** stigma, disclosure, dementia, psychosocial, post-diagnosis

## Abstract

**Objective:**

This study describes the adaptation of Honest, Open, Proud (HOP), to develop an empowerment intervention supporting disclosure decision-making for dyads of people living with dementia and their chosen supporter.

**Methods:**

Medical Research Council guidelines for developing complex interventions informed intervention development and feasibility testing. This included identifying the evidence base and theory (establishing HOP theory of change, a systematic review on decision-making in dementia, a stakeholder consultation), modelling the intervention materials with research experts (creation of version 1.0) and experts by experience (creation of version 2.0), and pilot testing the intervention recording participant observations and facilitator reflections. The final version of the intervention materials was developed with experts by experience of dementia where the accessibility of language and appropriate styles of facilitation were the focus.

**Results:**

The concept of the intervention was strongly endorsed by respondents of the stakeholder consultation (209/226). Stakeholder preferences included face-to-face delivery, a manualized workbook approach and the inclusion of the primary carer during intervention delivery. Recruitment for intervention groups took place in non-NHS settings (2 small groups recruited) and NHS settings (no groups recruited). In non-NHS settings, 7 dyads agreed to take part in one of two intervention groups. Both intervention groups had over 70% attendance by participants (group 1: 72.2% group 2: 87.5%).

**Conclusion:**

The concept of an intervention to support diagnostic disclosure was endorsed by stakeholders; however, recruitment was challenging; the “who to tell, how and when?” intervention has the potential to fill a gap in the post-diagnostic pathway.

## Introduction

Dementia is a syndrome affecting approximately 50 million people worldwide, with numbers set to rise to 131.5 million by 2051, it is characterized by a decline in and ultimately loss of cognitive functions such as memory, language and decision-making. Historically a diagnosis of dementia was often kept secret from the person with the condition. Nowadays, many people are being diagnosed with dementia at early stages. 1 Receiving a diagnosis of dementia or major-neuro-cognitive disorder is a life-changing transition.^2^ Dementia, to an extent in the early stages, is a concealable diagnosis and therefore individuals wishing to tell others have a series of decisions to make about whether to disclose. These may include, who, how and when to tell others about a diagnosis of dementia.

Knowing and being able to talk about one’s diagnosis can empower people to access services and support, to plan for the future, or become activists or advocates.^3^ On the other hand, many individuals in the early stages, whose symptoms are mild, worry about telling others, and how and when to tell them.^2^ Secrecy or dilemmas around disclosing can lead to harmful psychological and social consequences for those living with a stigmatized identity; however, secrecy can also provide protection from further stigmatization.^4^ Not knowing who, how or when to tell others about a diagnosis of dementia and associated difficulties can be disempowering, leading some people living with dementia, and their close family, to cut themselves off from social activities and pastimes.^2^ It is important to acknowledge the complexities of disclosure that are grounded in various contexts that may be dynamic and highly individualized.^5^

The capacity for people living with dementia to maintain daily obligations and fulfil their potential (concept of social health^6^) is influenced by social factors such as the presence or absence of a social network and the existence of stigmatization. The Social Health Taskforce of a pan-European research network for early timely and quality psychosocial interventions in dementia (INTERDEM) suggested focusing interventions around decision-making to protect and promote the competencies and rights of people living with dementia.^7^ An example of this would be a decision-making intervention that seeks to empower people in reaching decisions about disclosing a diagnosis of dementia. A recent INTERDEM manifesto issued a call for psychosocial interventions to promote dignity and autonomy through enhancing social integration for people living with dementia and their families.^8^

The effectiveness of complex interventions relies on robust design and development.^9^ Medical Research Council (MRC) guidelines for developing complex interventions for the public health sector describe the advantage of a rigorous development process, including maximizing effectiveness both in terms of cost and patient experience.^9^,^10^ The key elements of the MRC guidelines for development and feasibility include: (1) reviewing the evidence base and theory, (2) modelling process and (3) testing.^10^ The modelling process can provide important information about the intervention content and design, and can lead to refinement.^10^ Feasibility and piloting includes testing the acceptability of intervention procedures and the feasibility of recruitment and attendance. As intervention development is an iterative process that should be consistently informed by the production of new data, it is appropriate to collect data (eg, observations and reflections) that inform changes to intervention delivery or format within the developmental process.^10^–^12^

The Honest, Open, Proud (HOP)  is a peer group psychosocial program originally devised in the USA. It is delivered over three sessions that considers different aspects of disclosure to support individuals with mental health problems in reaching careful decisions concerning disclosure.^13^–^15^ The HOP program in its original form or adapted version has now been delivered in the USA, Australia, Belgium, Canada, Chile, China, Germany, Israel, Italy, Switzerland and the UK, to support disclosure decisions of mental health problems and other stigmatized conditions.^13^

This paper describes the development and preliminary feasibility testing of the “Who to tell, how and when?” intervention, an adaptation of HOP for people living with dementia, with a particular emphasis on co-production with people affected by dementia. The development and feasibility/piloting stages of the MRC framework^10^ were implemented as no specific guidelines were available for adapting HOP.

### Aims

#### Phase 1 (Development): Identifying the Evidence Base and Theory

The aim of phase one was to generate data to inform the dementia-specific adaptation of HOP by identifying the key tenets for HOP and understanding the challenges to decision-making in dementia.

#### Phase 2 (Development): Modelling Process

The aim of phase two was to adapt HOP to create a dementia-specific disclosure-decision-making intervention.

#### Phase 3: Feasibility Pilot

In phase three we sought to understand the feasibility of recruitment and delivery of the “who to tell, how and when?” intervention and understand potential avenues of improving intervention delivery.

## Methods

**Figure 1:**
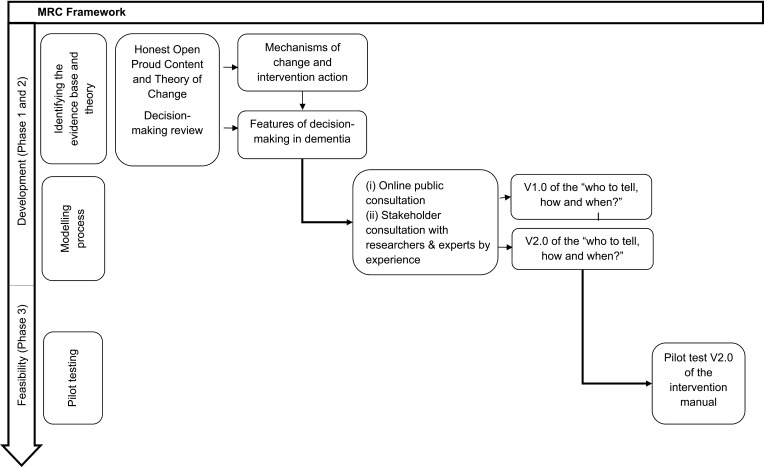
MRC framework for development and feasibility testing for complex interventions.

A schematic overview of the development and feasibility phases are shown in Figure 1. The development of the novel intervention began in November 2017. Non-NHS recruitment took place from December 2018 – February 2019 and NHS recruitment took place between September 2019 and December 2019.

### Phase One: Reviewing the Evidence Base and Theory

Existing HOP literature was collated and summarized to formulate a theory of change based on the empirical evidence presented in randomized controlled trials (RCTs) and cross-sectional studies. A recent systematic review^16^ was used to identify key features of decision-making processes for people living with dementia and understand how disclosure decision-making may take place in dementia.

### Phase Two: Modelling Process

The modelling phase of intervention development included (i) an online public consultation to gather preliminary evidence for stakeholder preferences followed by (ii) detailed consultations with the research team and experts by experience to co-production of intervention materials.

#### Online Public Consultation

HOP was originally designed for those with mental health diagnoses; therefore, it was necessary to identify dementia-specific preferences in design, content and engagement. Public opinions and preferences were collected through an online survey with three lines of enquiry. Firstly, to identify perceived barriers to disclosing a diagnosis of dementia, using multiple choice questions based on a psychosocial model of understanding the experience of receiving a diagnosis of dementia^17^ Secondly, to identify design preferences such as intervention and session length, delivery and format. Thirdly, to identify potential barriers and facilitators to engagement of people living with dementia with an intervention of this nature. The survey was set up on the Qualtrics online platform with the link to the survey disseminated through social media outlets (Twitter, Facebook), the Contact Help Advice and Information Network (CHAIN) and websites (UCL Division of Psychiatry, Alzheimer’s Society and UCL Unit for Stigma Research). There were no selection or screening procedures. As there were no incentives for completion, it was assumed that those completing the survey were people with some knowledge or, of interest in, dementia.

Questions for the stakeholder consultation were developed by the first author then reviewed by the Promoting Independence in Dementia (PRIDE) Public and Patient Involvement (PPI) group who made suggestions for changes to sentence structure to improve readability. They also suggested additional response options. The stakeholder consultation was conducted over a period of four months (November 2017 to February 2018). A copy of the survey is included in supplementary materials. All respondents saw the same set of questions, with space for optional free text. No personally identifiable or sensitive information (eg, demographics, health-related information) was collected, and there were no mandatory questions (ie, respondents could move through the survey leaving items unanswered).

#### Researchers and Expert by Experience Consultations

##### HOP adaptation within the research team (V1.0)

In the first instance, consultation within the research team (JB, TR, KS and GC) on the HOP adaptation took place focused on cultural adaptation, dementia-specific adaptation and readability; amendments or additions were made where necessary and version one (V1.0) of the intervention workbook was created.

##### Adaption for Culture and Participant Group

HOP was originally designed for a North American population with mental health diagnoses and therefore needed to be appropriate and relevant for a UK population of people affected by dementia, and for delivery to both people with dementia and their chosen supporter.

##### Readability

Changes to HOP were discussed through the lens of readability (the ease through which one can understand and decipher written text). For example, sentences longer than 20 words require greater reliance on memory and often have a complex syntax adding a further layer of difficulty.^19^ Readability was formally assessed using the readability statistics function in Microsoft Word 2016 such as the Flesch-Kincaid reading.^20^,^21^

##### Co-Production with Experts by Experience (V2.0)

Following consultations within the research team, with input from four carers of people living with dementia hereafter referred to as “experts by experience” (EbEs) version two (V2.0) of the workbook was created for preliminary feasibility testing. EbEs were members of an existing PPI group at University College London and one Research Network Member from the Alzheimer’s Society. A co-production meeting was held with EbEs and two members of the research team (JB and GC) over half a day. The structure of the meeting followed the chronological order of the workbook. The following questions were put to EbEs for each intervention section: (1) is this acceptable and suitable for people living with dementia; (2) what parts are good and from these which should be kept; (3) what should be changed, improved or removed from the manual. Based on the discussions with the EbEs, changes were made to the participant workbook and a facilitator’s guide was created.

### Phase Three: Preliminary Feasibility Testing

The “who to tell, how and when?” intervention (workbook V2.0 and facilitators guide V1.0) developed from Phase I and II was piloted in third (voluntary) sector settings and in the National Health Service (NHS). Participants were made up of dyads of people living with dementia and their chosen supporter (eg, a carer).

#### Recruitment and Eligibility

Join Dementia Research (JDR, https://www.joindementiaresearch.nihr.ac.uk/) an online register of volunteers who are interested in taking part in dementia research was used to recruit for groups in non-NHS settings. Attendance at memory clinics and referrals from clinicians in an outer London NHS Trust were strategies used to recruit in the NHS. Participant parameters were: adults over the age of 18; with a formal primary progressive diagnosis of dementia or a family carer or supporter of someone with such a diagnosis; ability to understand communicate, read and write in the English language; willingness to participate in the intervention and a follow-up interview. Participants were excluded if they did not have the capacity to give informed consent, if they were in the latter stages of a chronic terminal medical condition or, had a sensory impairment of such a severity that they would not be able to engage, or if they were expressing suicidal ideation or intent.

#### Ethics

Univeristy College London Research Ethics Committee [14001/001] and the NHS Surrey Borders Research Ethics Committee [19/LO/1163] granted ethical approval for this research.

#### Intervention Delivery

A consultant clinical psychologist and a trainee clinical psychologist co-facilitated group 1. The first author (PhD student, psychology masters graduate and former Alzheimer’s Society support worker) and a Dementia Wellbeing Lead based at Age UK facilitated group 2. All facilitators had experience of working with people affected by dementia. Each group underwent one intervention session a week (90 minutes) for a three-week period, delivered alongside the participant workbook.

#### Data Analysis

##### Participant Attendance

To calculate attendance feasibility, the mean was taken of the number of sessions each participant attended divided by the total number of sessions that could have been attended, this was then presented as a percentage.

##### Qualitative Observations and Facilitator Reflections

Qualitative observations were based on systematic descriptions of the intervention sessions (timing structure, delivery, content, practicalities) by TR in line with recent guidelines.^22^ Facilitator reflections were recorded by the observer immediately following each intervention session. Observations of the group and facilitator reflections were recorded with the aim to capture anything that could inform further intervention development such as ways to improve intervention delivery.

##### Qualitative Follow-Up Interviews

Participants who attended the intervention were also invited to take part in semi-structured, audio-recorded follow-up interviews, which were used to understand the experiences of participants who attended the group. These data are not presented in this paper.

## Results

### Phase One: Reviewing the Evidence Base and Theory

#### Honest Open Proud (HOP): Content and Theory of Change

The key tenets of HOP are that disclosing mental health problems is a personal decision and disclosure is an ongoing process where costs versus benefits of disclosure are often revisited depending on the context. HOP acknowledges both the positive and negative consequences of disclosure, encouraging participants to construct a personalized narrative of their mental health problems through a manualized, peer-supported format. The first session considers the pros and cons of disclosing, the second session explores the different ways to disclose and the final session is based on supporting participants tell their story in a meaningful way.

HOP seeks to support disclosure decisions and provide peer support to reduce the negative consequences of secrecy (stress or fear of being found out) and self-stigmatization whilst increasing levels of empowerment, self-efficacy in terms of coping with stigmatization, and aid participants towards optimal well-being and recovery.^13^,^15^ The aforementioned theory of change is partly supported by randomized controlled trials (RCTs) with adolescents and adults with mental health difficulties in the USA,^14^ Germany^23^ and Switzerland.^24^ RCTs suggest HOP is related to reductions in stress caused by stigma,^23^,^24^ reductions in self-stigma,^14^,^24^ decreased disclosure-related distress and perceived levels of secrecy,^23^,^24^ and an increase in intentions to seek help from family/friends and professionals.^23^

#### The Nature of Decision-Making in Dementia: A Systematic Review

A published systematic review into the nature of decision-making in dementia^16^ provided two vital points for consideration when adapting HOP for dementia. First, in order to create a meaningful decision-making environment for people living with dementia the Freedom of Choice factors (being informed, being listened to, ability to express opinions, time for reflection, and reversibility of choice) must be upheld but contextual factors can affect this (risk, relationships or resources).^25^ Secondly, the involvement of supporters (eg, carers, spouses, family members) can be both facilitative and disruptive to the decision-making involvement of a person living with dementia therefore contextual factors must be understood. Accordingly, dyadic adaptation of HOP was felt to be appropriate given the well-documented advantages of a dyadic approach, including positive effects of quality of life and cognition for people living with dementia, improved caregiver strain and psychological morbidity in caring spouses and improved relationship quality within the dyad.^26^–^28^ As the systematic review did not contain material on disclosure decision-making in dementia, a separate search was undertaken to identify disclosure decision models. Three were found but none were specific to dementia.^29^–^31^ All three considered the psychological risks of disclosure, and “third party decision-making” was mentioned which has relevance for the decision-making process for people living with dementia.^16^,^31^ Two of the disclosure decision-making models emphasized the role stigma can play in disclosure decision-making such that individuals with stigmatized labels (dementia) may choose secrecy as a way of avoiding public stigma.^29^,^30^ In order to better understand the views of people with dementia, family carers and the wider public on barriers to disclosure and on carer involvement, these issues were carried forward to the stakeholders’ consultations (phase 2).

### Phase Two: Modelling Process

#### Online Consultation

##### Respondents

A total of 226 people responded including people living with dementia (n=18), family carers of people living with dementia (n=85), health and social care workers (n=43), members of the general public (n=64), researchers (n=13) and others (n=3). The survey results are presented in Table 1. The free text responses were used to contextualize the numerical findings and are presented below in quotation marks.

**Table 1:** Summary of Stakeholder Consultation Results

Response Categories	PLWD (N = 18)	Family Carers (N = 85)	Health/Social Care Worker (N = 43)	Member of Public (N = 64)	Researcher/Academic (N = 13)	Other (N = 3)
**Barriers to Disclosure N(%)**						
Worry that others will view them differently (example, less able)	11(61)	60(71)	35(81)	52(81)	12(92)	3(100)
Shame	7(38)	23(27)	22(51)	21(33)	8(62)	3(100)
Unsure of what to say or what language to use	7(38)	25(29)	21(49)	18(28)	4(31)	2(67)
Not wanting to use the word “dementia”	5(28)	39(46)	29(67)	17(27)	7(54)	2(67)
Not knowing who to tell	7(39)	11(13)	14(33)	14(22)	7(54)	1(33)
Scared of what might be ahead	9(50)	54(64)	27(63)	38(59)	10(77)	3(100)
Talking about it makes it more real	6(33)	44(52)	25(58)	35(55)	6(46)	2(67)
Not accepting/denying the diagnosis	3(17)	40(47)	30(70)	29(45)	10(77)	3(100)
Worry about losing relationships	5(28)	15(18)	24(56)	16(25)	8(62)	1(33)
Worry that others may avoid or exclude them	9(50)	36(42)	27(63)	28(44)	11(85)	1(33)
Not wanting to burden or upset others	8(44)	50(59)	28(65)	48(75)	8(62)	3(100)
Carer or family not wanting them to tell others	4(22)	13(15)	20(47)	13(20)	8(62)	1(33)
Other	2(11)	7(8)	3(7)	2(3)	1(8)	1(33)
**Preferred Delivery Method N(%)**						
Face to Face	13(72)	74(87)	30(70)	53(83)	10(77)	2(67)
Self-Guided	8(44)	21(25)	12(28)	13(20)	6(46)	1(33)
Other	4(22)	15(18)	11(26)	8(13)	5(38)	1(33)
**Barriers to Engagement N(%)**						
Not knowing enough about dementia	9(50)	18(21)	14(33)	23(36)	4(31)	1(33)
Embarrassment	14(78)	55(65)	31(72)	42(66)	7(55)	3(100)
Wanting to “keep it in the family”	10(56)	55(65)	32(74)	28(44)	8(62)	2(67)
Not wanting “outside help”	7(39)	62(73)	31(72)	38(59)	10(77)	2(67)
Fear of diagnosis	7(39)	47(55)	37(86)	35(55)	6(46)	3(100)
Wanting to keep the diagnosis to themselves	10(56)	42(49)	31(72)	28(44)	10(77)	2(67)
May have other ways of deciding who to tell, how and when	6(33)	15(18)	15(35)	7(11)	8(62)	1(33)
Not knowing the programme exists	10(56)	65(76)	37(86)	53(83)	10(77)	2(67)
Worrying about travelling (if it is a group programme)	6(33)	34(40)	28(65)	22(34)	9(69)	1(33)
Not wanting to be in a group with other people who have dementia	10(56)	56(66)	29(67)	27(42)	11(85)	2(67)
Other	0(0)	3(4)	6(14)	3(5)	3(23)	1(33)
**Facilitators to Engagement N(%)**						
Previous knowledge about dementia	8(44)	24(28)	10(23)	20(31)	4(31)	0(0)
Support from their family or friends	15(83)	79(93)	39(91)	49(77)	12(92)	3(100)
Trained facilitator with personal experience of dementia	16(89)	59(69)	35(81)	48(75)	8(62)	2(67)
More information to help them decide if it is for them	10(56)	44(52)	28(65)	33(52)	10(77)	1(33)
Group delivery to take place outside clinical settings (eg community centre)	8(44)	35(41)	30(70)	25(39)	9(69)	0(0)
Built-in involvement of primary supporter	8(44)	42(49)	18(42)	25(39)	9(69)	1(33)
Other	3(17)	3(4)	5(12)	2(3)	2(15)	0(0)

##### Barriers to Disclosure

The survey provided evidence that all categories of respondent believed that there are barriers to people with dementia disclosing their diagnosis to others. There were differences between responders in different categories All respondents rated “worry that others will view them differently (eg, less able)” as the top barrier to disclosing a diagnosis of dementia (Table 1). People living with dementia also rated the following as dominant barriers: scared of what might be ahead; worry that others may avoid or exclude them; not wanting to burden or upset others (“feeling a failure to my family, that I had let them down”); shame (“people saying ‘don’t be silly there is nothing wrong with you’”); unsure of what to say or what language to use and not knowing who to tell. The endorsement of the latter two barriers helped to build the rationale for a disclosure decision-making intervention as language and planning who to tell were existing tenets of HOP. The loss of independence as a barrier to disclosure was noted in additional text comments by carers (“losing independence”), members of the public (“fear of losing driving license”) and researcher and academics (“concern about any effect on their employment”).

##### Preferences for Delivery

Regarding method of delivery, face-to-face rather than self-guided was preferred across all respondent groups. Examples of “other” responses included a mixture of face to face and self-guided delivery (“perhaps a combination of the two, some face to face and some self-guided”). Of alternative face-to-face delivery approaches, respondents living with dementia preferred delivery in small groups (“a group discussion would be good to have more thoughts towards the discussion”) where survey respondents mentioned in additional text comments that carers should also attend. Concerning session length, respondents unanimously preferred one session a week for a three-week period with sessions lasting one to 1 ½ hours over other options (full day workshop, or two half days). All survey respondents acknowledged in the free text comments that “flexibility is key” and it depended on the preferences of the person living with dementia.

##### Facilitators and Barriers to Engagement

People living with dementia identified the following barriers to engagement with the proposed intervention: embarrassment, wanting to “keep it in the family”; wanting to keep the diagnosis to themselves; not wanting to be in a group with other people who have dementia; and not knowing enough about dementia. In text comments people living with dementia noted the “fear of doing something new”, “lack of insight into diagnosis” and “not believing the diagnosis” were barriers to intervention engagement. People living with dementia endorsed the following facilitators to engagement: support from their family or friends; more information to decide if the program is for them; built in involvement of primary carer; and groups to take place outside of clinical settings. One person living with dementia mentioned that it is “difficult to encourage people with dementia to reach out. More options the better” and one carer noted that facilitators may want to conduct a “home visit … [for the person living with dementia] to have a friendly face for the first session”. Similar to the systematic review findings,^16^ several respondents spoke to the importance of including carers (“shared experiences make it easier and stop the feelings of isolation. Carers should also attend”).

In summary, a large majority of respondents agreed that people who are diagnosed with dementia would benefit from an intervention designed to support disclosure decisions (209/226). In line with the findings from the stakeholder consultation, a 3 x 90 minute group intervention was planned for dyads of people living with dementia and their carer to be delivered outside of clinical settings.

#### Researchers and Expert by Experience Consultations

##### HOP Adaptation Within the Research Team (Creating V1.0)

###### Cultural Adaptation

To avoid potential negative interpretations of the terms “Honest”, “Open” and “Proud” (eg, suggestions that someone is dishonest or not proud if they do not disclose), the title was changed to “who to tell, how and when?”. References in HOP to “coming out” were replaced with “telling” as, at least in the UK, the term “coming out” is still heavily associated with sexuality disclosure. Furthermore, the purpose of the intervention was not to promote “coming out” but rather to empower participants to reach decisions about disclosure themselves. The original outline of HOP was retained such that each session covered similar content however the adaptation was grounded in the context of dementia, see Table 2.

**Table 2:** Comparison Between Original HOP and “Who to Tell, How and When?” Adaptation for People Living with Dementia

Honest Open Proud			“Who to Tell, How and When?”	
**Session TitleContents**		**Session Title Contents**	
**Considering the pros and cons of disclosing:**	The stories we tell ourselves/identify beliefs participants hold about themselves; Identifying hurtful and helpful attitudes about mental illness; Challenge personally hurtful beliefs; Weigh pros and cons of disclosure to facilitate a decision on whether to disclose.	**Session 1**	**Talking about dementia**	Talking about dementia – what’s in a name? What might a diagnosis mean for a person’s sense of “who they are” and their outlook on life? What are the advantages and disadvantages of telling or not telling others
**Different ways to disclose:**	Different ways to disclose and weighing the pros and cons of each; Selecting a person to whom one might disclose; Consider how others might respond to a disclosure and how their response might affect one’s self.	**Session 2**	**Who to tell, how and when?**	Different ways to tell others Who already knows and who in your life do you want or may want to tell. Who are you unsure about and must not be told How and when to tell others? What may the reactions of others be?
**Telling your story**	How to tell one’s story in a personally meaningful way; Review how telling one’s story went; Peer support for disclosure; Put together all that’s been learnt in order to move forward.	**Session 3**	**Support for me, for you, for us**	Sharing experiences of telling others Planning to tell someone (who, how and when?) Whose diagnosis is it?* When other people do the telling* Where may you find sources of support*

**Notes:** *Elements unique to “Who to tell, how and when?”.

###### Dementia-Specific Adaptation

Throughout the HOP workbook, “mental illness” was replaced with “dementia”. To ensure examples were grounded in real-life experiences of people living with dementia, qualitative data from the PRomoting Independence in DEmentia (PRIDE) intervention manual was used to develop suitable examples.^18^ Examples in the original HOP workbook (eg, advantages and disadvantages of disclosing a diagnosis of schizophrenia) were changed to be dementia-specific. For example, short quotes were used in the workbook to communicate possible advantages (eg, “When I get muddled with change at my local shop, the shop keeper reaches over to help me … It relaxes me that he knows”; workbook p10) and disadvantages (eg, “After telling my family, I have been feeling that people have put me down. They don’t listen to my opinion”; workbook p11) of sharing a dementia diagnosis.

###### Dyadic Adaptation

Care was taken so that wording could relate to both a person living with dementia and their chosen supporter. Hence, personal pronouns that spoke directly to a person with the diagnosis were removed. The workbook examples aimed to speak to the dyad’s respective dementia disclosure experiences alongside discursive exercises designed to facilitate communication between the dyad and within the group around the issue of dementia disclosure.

###### Readability

Complex sentences were removed, front size was increased and changed to a sans serif style, the HOP workbook was condensed and content removed which appeared in a “facilitator’s guide” instead so this could be covered verbally. All essential information was kept such as session objectives, sub-section introductions, and task objectives and embedded worksheets as it was necessary for the workbook to flow as a standalone document if attendees were to read it outside of group sessions. In comparison to the original HOP manual all readability statistics improved in the “who to tell, how and when?” workbook (Table 3).

**Table 3:** Summary of Readability Statistics for the Honest, Open, Proud Program and the “Who to Tell, How and When?” Dementia Adaptation

Readability Domains	Honest, Open, Proud, Program (N)	The “who to Tell, How and When?” Intervention (N)
Words	25,126	2542
Characters	124,461	14,034
Paragraphs	1130	217
Sentences	1576	135
Sentences per paragraph	2.7	1.9
Words per sentence	14.3	12.4
Characters per word	4.7	4.4
Passive sentences	8%	3%
Flesch reading ease^a^	60.6	71.3
Flesch-Kincaid grade level^b^	8.2	6.4

**Notes:** ^a^Score metric between 0 to 100, higher scores indicated greater readability. ^b^Equivalent to United States grade level of education.

##### Co-Production with Experts by Experience (Creating V 2.0)

###### Participant Workbook

Language changes were recommended by EbEs such as using the terms “advantages and disadvantages” rather than the HOP wording of “costs and benefits” when weighing up whether or not to disclose a diagnosis. Further, in the first session, when language and its potential impact on a person’s identity is discussed, EbEs felt that “identity” was very abstract and that the term “outlook” was preferable as it encompassed behavioral effects as well as emotional and psychological consequences of the diagnosis. In the original HOP, manual tables were used for exercises, for example, a table where participants can list the ‘costs and benefits’ of telling others about a diagnosis. EbEs were of the opinion that these should be replaced by notes sections as tables can be difficult to navigate and force contributions more so than a blank notes section alongside a meaningful conversation. EbEs generally liked the examples in the booklet; however, they recommended that when more than one person was included in an example that they were of different genders with names that sounded different so as not to confuse participants when the example was discussed.

###### Facilitator’s Guide

EbEs endorsed the idea of a facilitator’s booklet to go alongside the participant version. They felt sensitivity to the potential harm language can do was of prime importance. For this reason, the facilitator booklet avoided any negative language around dementia such as “sufferer” or “patient”. The term “caregiver” was contested during our discussions and therefore the term “supporter” or “carer” was used. Although EbEs were content with the session summaries, they requested the facilitators ask if participants wanted to cover specific topics in the next session. This was key to making the intervention as person-centered and individualistic as possible. EbEs emphasized that the role of the facilitator should not just be to deliver the intervention but also to perform a “signposting” role supplemented by the “sources for support” page at the end of the booklet.

In summary, unlike HOP, which is peer-led, the intervention adapted for dementia was designed to be delivered by facilitators skilled in working with people affected by dementia, such as, Admiral Nurses, Age UK employees and trained Alzheimer’s Society volunteers. Another fundamental difference in format between HOP and “who to tell, how and when?” is the inclusion of a chosen supporter or carer during the intervention sessions. As a carer is seen in their own right as a participant and often shares the effects of adementia diagnosis, changes in the language of the intervention materials were made and novel topics introduced, such as “whose diagnosis is it” to reflect this. Many features of HOP were still implemented in the “who to tell, how and when?” intervention, such as weekly sessions over a three-week period and the notion of a manualized approach for participants to follow.

### Phase Three: Preliminary Feasibility Testing

#### Feasibility of Recruitment and Attendance

##### Non-NHS Settings Recruitment

**Figure 2:**
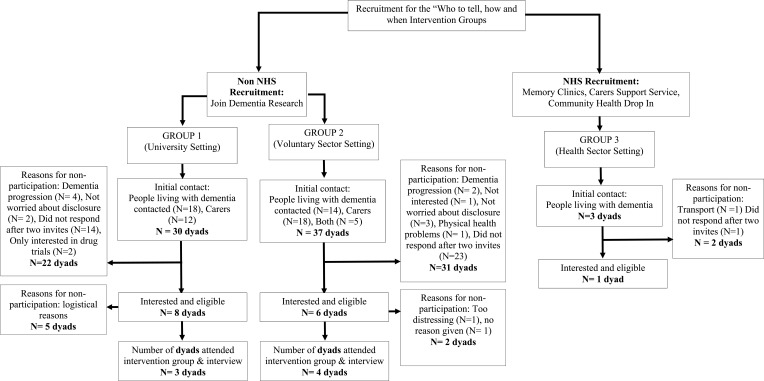
Recruitment and attrition of participants attending the “who to tell, how and when?” intervention.

Sixty-seven dyads in total were identified of which 14 dyads were eligible, interested, responded to study invitations and took part in non-NHS settings in two smaller groups (group 1: 8 dyads; group 2: 6 dyads). For group 1, 5 dyads were not able to take part, for group 2, 2 dyads were not able to take part, see Figure 2 for reasons for non-participation. Both intervention groups had over 70% attendance (group 1: 72.2%, group 2: 87.5%; see Table 4).

**Table 4:** Participant Characteristics for “Who to Tell, How and When?” Intervention Groups

Sociodemographic Characteristics	Group 1		Group 2	
	PLWD	Carers	PLWD	Carers
Age, years Mean (SD)	77.22 (11.55)	71.33 (8.37)	72.52 (5.94)	72.31 (2.47)
Gender (M/F)	1/2	1/2	2/2	2/2
Months since diagnosis	16.66	–	25.75	–
Type of dementia				
Alzheimer’s Disease	2	–	4	–
Vascular Dementia	1	–	0	–
Relationship between PLWD and carer				
Spousal	2		4	
Other	1		0	
Ethnicity				
White	2	3	4	3
Other Ethnic Group	1	0	0	1
Participant Session Attendance				
All Sessions	2	1	3	3
Two Sessions	1	0	0	1
One Session	0	2	1	0

**Abbreviation:** PLWD, people living with dementia.

##### NHS Recruitment

The NHS group did not take place as a there were too few participants for a group to start, reasons for non-participation are presented in Figure 2 and barriers to recruitment in NHS settings will be discussed in the next section based on records kept by the NHS recruiting site and feedback from Clinicians working within the recruiting NHS Trust.

#### Qualitative Observations and Facilitator Reflections

##### Group 1

###### Qualitative Observations

Planned intervention content was delivered; however, facilitators had to repeat material to accommodate non-attendance. The liveliest discussion was linked to an exercise within the workbook that required independent work followed by paired and group discussions. It became evident that carer-specific examples were missing from the workbook.

###### Facilitator Reflections

Participants in this group had diverse perspectives about their diagnosis (eg, one facilitator noted that “one person with dementia who, by their own admission, was strongly avoidant”, and another who was “struggling with the aftermath of being told their diagnosis in an insensitive fashion”). In common with the independent observer, facilitators noted the value of independent/paired work in preparation for group discussions.

###### Recommendations from Group 1 Implemented in Group 2

Setting aside “arrival time” for refreshments and informal socializing; more explicit statements of session aims and exercise aims; use of visual prompts and written materials that could be used during the session; varying the discussion format varied (pairs, small groups, and whole group).

##### Group 2

###### Qualitative Observations

All intervention content was delivered for each session. Participants wished to discuss their experience of receiving their diagnosis (not explicitly included in the workbook). Written support strategies were missing in the workbook.

###### Facilitator Reflections

Changing the delivery format of exercises (eg, between small and large group discussion) facilitated involvement from quieter participants and increased communication between and within dyads. Pre-session socialization built rapport.

###### Recommendations for Future Groups

Carer-specific quotes should be included in the workbook. Space to discuss the experience of receiving a diagnosis should be provided along with more detailed support strategies to deal with the negative reactions of others.

## Discussion

This paper describes the development, and preliminary attendance feasibility evaluation, of an empowerment intervention to support people affected by dementia make decisions around disclosing a diagnosis. The “Who to tell, how and when?” intervention was field-tested in non-NHS settings as a dyadic, group-based, manualized intervention led by a trained facilitator, following a three-stage development and testing process where the views of those affected by dementia informed design and delivery features.

### In the Context of Current Post-Diagnostic Support Services

Although variation exists in attitudes to dementia and the services available to those affected, it can be agreed that, in order for post-diagnostic support services to be accessed, people living with dementia are often required to meet the standard of being able to talk about their diagnosis.^1^,^32^ Therefore, in the face of a life changing, stigmatized diagnosis, people living with dementia and carers are often left to negotiate decision-making around telling others in their social networks about the diagnosis, with no post-diagnostic support in place.^2^

The recent emphasis on promoting the social health of people affected by dementia calls for timely interventions to empower decision-making to maintain social networks.^8^ Improvement in psychological well-being for both partners in the dyad improved quality of life, and increased knowledge of one another’s coping skills, have been found by previous dyadic interventional studies, thus providing an evidence base for a dyadic psychosocial approach over more individualized interventions.^28^ Together, the literature suggests an important gap in the diagnostic pathway that can be filled with an empowerment based approach-supporting dyads affected by dementia. The “Who to tell, how and when?” intervention is the first empowerment intervention to support disclosure decision-making in people affected by dementia, and was endorsed by the majority of respondents in the online stakeholder consultation.

Qualitative observations and facilitator reflections were recorded and used as per the MRC process evaluation guidance to understand how the intervention was implemented.^33^,^33^ Changing the format of activities was a powerful tool to involve all participants.

### Strengths and Limitations

The benefit of a rigorous development and feasibility procedure, as outlined by the MRC framework, is that intervention materials can be developed and tested to maximize any worthwhile effect and foresee implementation issues before potential examination in a full-scale trial. This is recommended by the MRC to minimize the later problems of acceptability, intervention delivery, recruitment and attendance. Speaking to the importance of rigorous development, the involvement people affected by dementia (the online stakeholder consultation, intervention production), increases intervention validity, such that materials are more likely to be grounded in the values of the target population. The key strength of this study is the involvement of people living with dementia and family carers. The results of the stakeholder consultation highlighted the importance of having the choice of people living with dementia rather than assuming that carers are an adequate proxy. However, it is important to acknowledge that EbEs who co-produced intervention materials were all carers rather than people living with dementia and therefore future iterations may benefit from the inclusion of people living with dementia in the co-production process.

Organizational and individual factors may have contributed to the lack of recruitment through the NHS. For example with a recent push for diagnoses, memory services work on an “assess, diagnose, discharge” model. Firstly, this means researchers typically meet potential participant’s immediately following diagnosis, which does not leave enough time for someone to have become worried or fearful about telling others about dementia; secondly, clinicians are often not able to get to know their patients enough to discuss the benefit of taking part in the “who to tell, how and when?” intervention. Clinicians who gave feedback about the intervention said that the intervention would be a valuable asset to post-diagnostic support, particularly as some clinicians also acknowledged that they had not had conversations with patients around whether they were worried about telling others. In terms of individual factors, the target group for the “Who to tell, how and when?” intervention may be harder to reach in comparison to other dementia-related psychosocial interventions. The stakeholder consultation previously presented highlighted several barriers to disclosure of a dementia diagnosis and also barriers to intervention engagement, collectively this may have led to low recruitment. For example, if potential participants were indeed fearful or worried about telling others as suggested by the stakeholder consultation results, they might be reluctant to attend a group-based intervention that explores fears and worries around telling others. In addition, many people attending memory services for a diagnosis often have other health conditions that require more attention or have a greater impact on daily life including psychological well-being; hence, a diagnosis of dementia may not be the most concerning diagnosis for some.

Non-NHS intervention groups were located in central London with good transport links however the NHS site used to recruit for this study was located in the outer London area with reduced transport links but also a larger population of ethnic minority communities particularly of South Asian origin whom may have more specific or differing barriers to disclosure and engagement.

Whilst several stages informed the “who to tell, how and when?” intervention, this process does not guarantee recruitment feasibility across settings and different participant characteristics (eg, ethnic minority groups, types of dementia), particularly, given the nuanced target population we sought to recruit. The majority participants recruited for both intervention groups identified as white, therefore questions still remain around whether the “who to tell, how and when?” intervention would benefit members of other ethnic groups.

During the feasibility-testing phase, no formal outcome measures were taken as the specific rationale for testing was to explore the feasibility of recruitment and retention. The “Who to tell, how and when?” intervention was adapted from HOP, a mental health intervention; therefore, it is important to consider whether both interventions have the same theory of change and underlying causal mechanisms. Due to the clinical differences between mental health and dementia, the theory of change in a dementia-related audience might differ. For example, the “Who to tell, how and when?” intervention may improve levels of empowerment indirectly through reducing decisional conflicts rather than reducing self-stigmatization. Therefore, decisional conflict or peer-support-related concepts may be better-fit primary outcomes based on the body of empirical work around decision-making in dementia in comparison to the very little work done in the dementia-related self-stigma field.^16^,^34^

### Future Directions

With regards to future recruitment, researchers attending pre-existing groups in non-NHS settings (eg, peer support or voluntary sector organized activities) to build relationships with potential participants may prove more fruitful than using an online approach; further recruiting a more ethnically diverse population will help to understand the transference of the “who to tell, how and when?” intervention.

For future recruitment in NHS settings, it is important to focus on recruiting potential participants during follow-up visits rather than after diagnostic interviews and focus efforts within primary care (eg, GPs). Additionally, speaking to clinicians beforehand to encourage them to ask whether patients are worried or fearful of telling others may lead to an increase in referrals. Lastly, it may be plausible to integrate the “Who to tell, how and when?” intervention into existing infrastructures such as post-diagnostic groups which are already run by some memory services. It may also be useful to feasibility test recruitment in NHS settings in other geographical areas.

Although face-to-face delivery was the preferred method from the results of the online stakeholder consultation, other delivery formats were less popular, but still selected by respondents. For this reason, it may be necessary for future testing to consider alternative forms of delivery (self-guided, remote facilitation, combinations of face to face and self-guided) to accommodate participants who do not wish to attend a group but would benefit from engaging with the intervention content.

As this is the first empowerment intervention of its kind, it is necessary to field test it further to understand the universality of the preferences generated by the stakeholder consultation and also the underlying causal mechanisms of action. Analyzing qualitative interview data from follow-up interviews will help to understand participant experiences of the intervention and can also be used to make iterative changes to intervention content, format and delivery, and inform future testing of suitable outcomes measures decreasing the chances of implementation error.^33^,^35^

## Conclusion

Honest, Open, Proud was adapted to form the “Who to tell, how and when?” intervention, an empowerment-based, dyadic, decision-making intervention to support people affected by dementia through diagnostic disclosure. Based on the results of the pilot study, the intervention groups were feasible in terms of participant attendance in non-NHS settings; however, recruitment figures were low for non-NHS settings and difficulties encountered in NHS settings resulted in no intervention groups taking place. The next step in the process of development of this complex intervention is to evaluate the acceptability of the “who to tell, how and when?” intervention and implement recommendations from the findings of the current study in future intervention groups.
